# Giant endometrial polyp in postmenopausal women without hormonal exposure: a case report and literature review

**DOI:** 10.3389/fmed.2026.1821347

**Published:** 2026-04-20

**Authors:** Danlin Wang, Li Wang, Ning Zhou, Yan Fang

**Affiliations:** Department of Gynaecology, Tongren People's Hospital, Tongren, Guizhou, China

**Keywords:** giant endometrial polyps, hysteroscopic, polypectomy, postmenopausal bleeding, total abdominal hysterectomy

## Abstract

**Objective:**

Giant endometrial polyps (EPs) are rare in postmenopausal women without hormonal exposure. This study aimed to summarize their clinical presentation and management.

**Patient:**

Fifteen postmenopausal women with primary giant EPs were included: one case from our center and fourteen additional cases were retrieved from Pubmed, Web of science and Embase databases.

**Results:**

The fifteen patients ranged in age from 55 to 70 years, with polyp diameters ranging from 4.0 to 12.0 cm. The most common symptom was postmenopausal bleeding (PMB). Surgical intervention was performed in all cases: six patients underwent hysteroscopic polypectomy, eight received total abdominal hysterectomy with bilateral salpingo-oophorectomy (TAH-BSO) and one underwent dilation and curettage D&C. Most polyps (11/15) were benign on histology and exhibited cystic dilatation.

**Conclusion:**

Giant EPs in postmenopausal women are rare but predominantly benign, especially in the absence of hormonal exposure. Transvaginal sonography (TVS) is the first-line modality for initial evaluation. Diagnostic hysteroscopy with complete polypectomy remains the gold standard, enabling accurate histopathological assessment and avoiding unnecessary hysterectomy.

## Introduction

Endometrial polyps (EPs) arise from localized overgrowth of endometrial glands and stroma. They represent one of the most common benign causes of abnormal uterine bleeding (AUB). The prevalence of EPs among women presenting with AUB ranges from 10 to 40% (1, 2). Notably, second only to endometrial atrophy, EP is a leading etiology of postmenopausal bleeding in postmenopausal women, and often warrants prompt evaluation because of the risk of underlying endometrial malignancy.

Most EPs are benign; however, malignant transformation can occur, and its risk appears to increase with advancing age (3). Atypical polyps, often arising in the context of concomitant endometrial hyperplasia, are more frequently observed in women with established risk factors for endometrial pathology (4). These risk factors for malignancy among EP patients include advanced age, obesity, hypertension, postmenopausal status, and tamoxifen use (5).

Tamoxifen, a selective estrogen receptor modulator, inhibits tumor cell proliferation in breast tissue through competitive antagonism of the estrogen receptor. However, it exerts a partial estrogen-agonistic effect on the endometrium, promoting endometrial proliferation and polyp formation (6). EPs are typically less than 2 cm in size, and those measuring greater than 4 cm are classified as giant EPs (7). These larger lesions occur more frequently in the context of unopposed estrogen stimulation, particularly in women receiving tamoxifen therapy for breast cancer (6). Although giant EPs are most commonly associated with hormone agents, they can also occur sporadically in postmenopausal women without hormonal exposure. These cases may mimic malignancy because of their size and presentation, leading to unnecessary aggressive surgery.

In this study, we present a case of a giant EP in a postmenopausal woman without hormone exposure and perform a literature review to summarize the clinical characteristics and management of similar patients. This case report and literature review are reported in compliance with the CARE guidelines (8).

## Case presentation

A 68-year-old woman (gravida 6 para 6) was admitted to a local hospital with postmenopausal spotting for 1 day. She was overweight (BMI 29.4 kg/m^2^) with no history of hypertension or diabetes. The patient had been postmenopausal for 17 years and denied any use of medications, particularly hormone agents. Transvaginal ultrasound (TVS) revealed an intrauterine heterogeneous mass measuring approximately 4.3 × 3.6 × 2.1 cm, while the surrounding endometrial thickness was only 2 mm. Fractional dilation and curettage were performed, but no endometrial tissue was obtained. Subsequently, biopsy forceps were used to obtain fragments of the intracavitary lesion under ultrasound guidance, and histopathological examination confirmed the diagnosis of an endometrial polyp.

**Figure 1:**
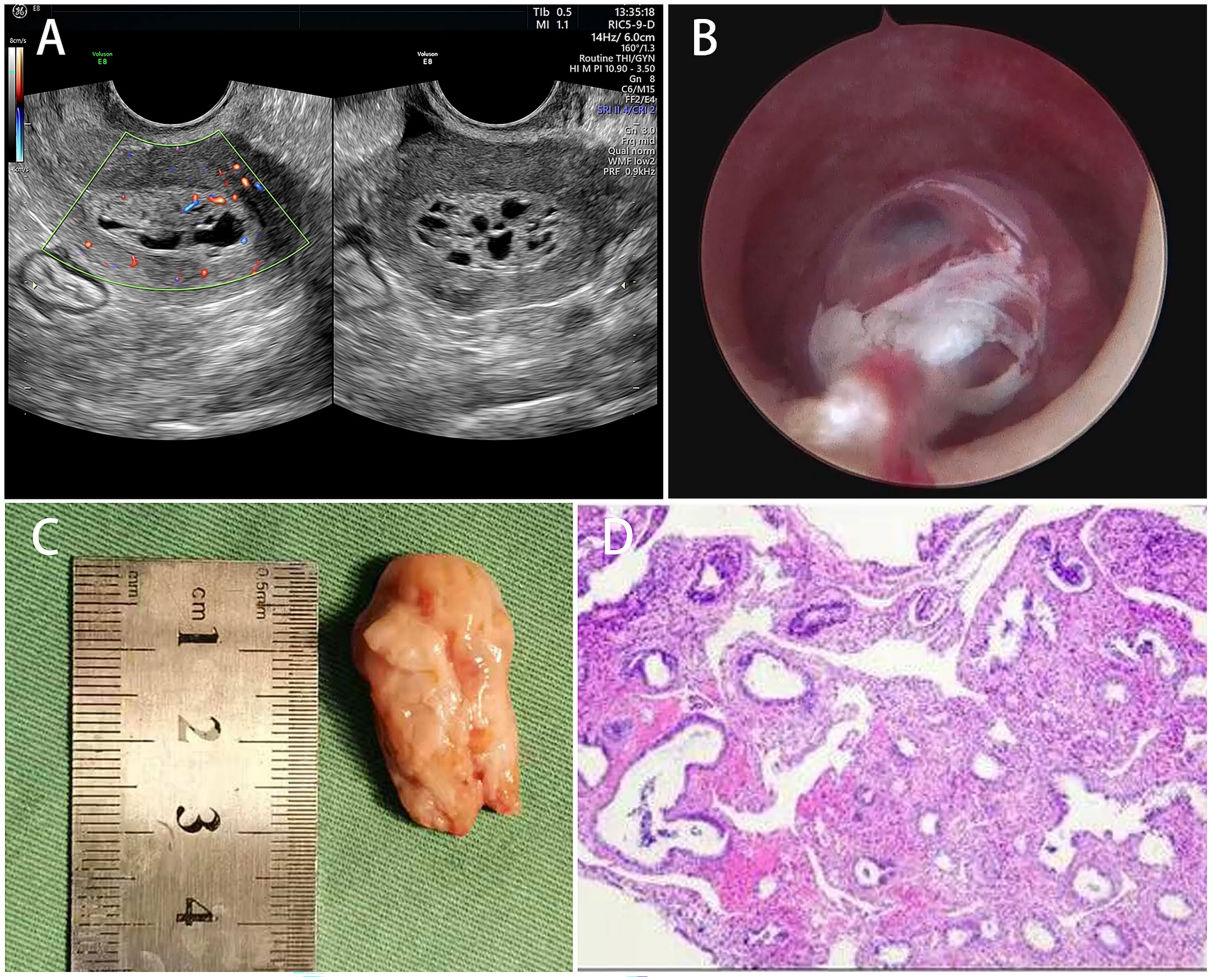
**(A)** Transvaginal ultrasound revealed a well-demarcated, heterogeneous intracavitary mass with honeycomb-like anechoic areas and rich vascularity. **(B)** Diagnostic hysteroscopy revealed a pedunculated intracavitary polyp arising from the posterior-right uterine wall. **(C)** Gross specimen after hysteroscopic polypectomy. **(D)** Histopathological analysis confirmed a benign cystic endometrial polyp without hyperplasia or malignancy.

For further evaluation and management, she was referred to our institution. Hormonal assessment revealed a FSH concentration of 30.50 mIU/mL and an E₂ concentration of 12.20 pg./mL, which is consistent with postmenopausal status. In addition, the serum CA125 concentration was 6.81 U/mL. TVS revealed a well-demarcated, heterogeneous intracavitary mass measuring 3.3 × 3.1 × 1.5 cm, containing multiple anechoic areas arranged in a honeycomb pattern. Notably, rich vascularity was observed in both the peripheral and solid components of the lesion, raising concern for endometrial carcinoma or submucosal leiomyoma (Figure 1A).

After informed consent was obtained, diagnostic hysteroscopy was performed. A pedunculated lesion approximately 3.0 × 3.0 × 1.5 cm in size was identified in the uterine cavity. The surface was smooth, pink, and soft, with multiple translucent cystic vesicles. The stalk originated from the posterior-right uterine wall without signs of neovascularization (Figure 1B). The polyp was completely resected using cold-knife hysteroscopic excision (Figure 1C). The procedure was uneventful. Histopathological analysis confirmed a benign cystic endometrial polyp, with no evidence of hyperplasia or malignancy (Figure 1D). The patient recovered well and was discharged on postoperative Day 2. Appropriate written consent for publication was obtained.

## Review of the literature

Relevant cases were retrieved from the PubMed, Web of Science and Embase databases to supplement the clinical context. The final literature search was performed in February 2026. Two independent reviewers screened eligible publications on the basis of the title and abstract content. The search strategy was as follows: ((Endometrial Polyp* or Uterine polyp* or Endometrial polypi) and (Postmenopause or Postmenopausal or Menopause)). All case reports describing giant EPs originating in the uterine cavity in postmenopausal women without prior hormonal exposure were included.

**Figure 2:**
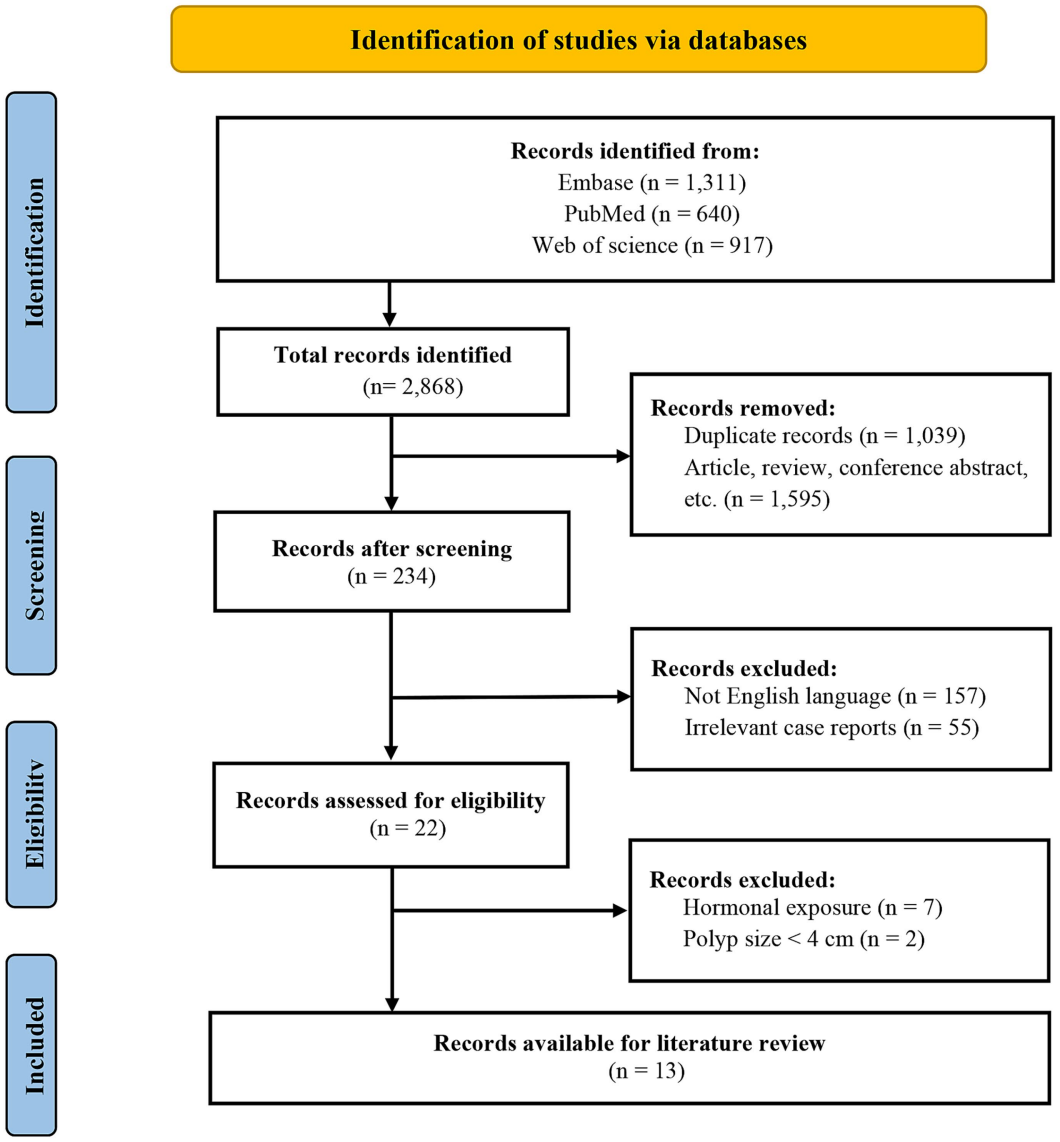
PRISMA flow diagram illustrating the literature search and selection process.

The study selection process is outlined in the PRISMA flow diagram (Figure 2). Finally, fourteen cases were identified from the above databases (7, 9–20). Therefore, a total of fifteen postmenopausal women with primary giant EPs were included. Their clinicopathologic features are summarized in Table 1. The patients ranged in age from 55 to 70 years, with polyp diameters ranging from 4.0 to 12.0 cm. The most common symptom was postmenopausal bleeding (PMB) (11/15). All lesions were managed surgically: six patients underwent hysteroscopic polypectomy, eight underwent total abdominal hysterectomy with bilateral salpingo-oophorectomy (TAH-BSO) and one underwent dilation and curettage (D&C). Notably, most polyps (11/15) were benign on histology and exhibited cystic dilatation, whereas the other four were malignant (two carcinomas, one carcinosarcoma, and one sarcoma).

**Table 1:** Clinicopathological features of postmenopausal women with giant endometrial polyp.

Case	Year	Age (year)	Initial presentation	Polyp size (cm)	Treatment	Cystic dilatation	Malignancy
Case 1 [11]	2013	66	PMB	12.0 × 6.0	TAH-BSO	Yes	No
Case 2 [12]	2014	70	Asymptomatic	10.0 × 9.5	TAH-BSO	Yes	No
Case 3 [13]	2017	65	Asymptomatic	8.5 × 1.5	Hysteroscopy	Yes	No
Case 4 [15]	2017	65	Asymptomatic	9.0 × 2.5	Hysteroscopy	Yes	No
Case 5 [15]	2017	60	PMB	8.0 × 4.0	Hysteroscopy	Yes	No
Case 6 [16]	2018	57	PMB	5.3 × 4.0	TAH-BSO	No	Carcinosarcoma
Case 7 [17]	2018	67	PMB	7.0 × 5.0	TAH-BSO	Yes	Sarcoma
Case 8 [7]	2018	58	PMB	5.0 × 4.0	Hysteroscopy	Yes	No
Case 9 [18]	2018	70	PMB	5.3	D&C	Yes	No
Case 10 [9]	2020	55	Asymptomatic	4.0 × 1.2	Hysteroscopy	No	No
Case 11 [19]	2020	67	PMB	7.0	TAH-BSO	No	Carcinoma
Case 12 [14]	2021	67	PMB	7.0 × 4.0	TAH-BSO	No	No
Case 13 [20]	2022	69	PMB	4.2	TAH-BSO	Yes	Carcinoma
Case 14 [10]	2024	59	PMB	5.5 × 3.0	TAH-BSO	Yes	No
Case 15	2026	68	PMB	4.3 × 3.6	Hysteroscopy	Yes	No

PMB, postmenopausal bleeding; TAH-BSO, total abdominal hysterectomy and bilateral salpingo-oophorectomy; D&C, dilation and curettage.

## Discussion

EPs are benign lesions composed of a variable proportion of endometrial glands, fibroblast-like spindle cell stroma, and thick-walled blood vessels and affect approximately 25% of women (21). Clinically, they most commonly present with AUB, followed by lower abdominal pain (22). Notably, EPs are a frequent cause of vaginal bleeding during the perimenopausal period. In this study, eleven of the fifteen patients presented with PMB, while the other four were asymptomatic and were diagnosed incidentally during routine gynecological examinations.

The ages of the fifteen patients ranged from 55 to 70 years, with polyp diameters ranging from 4.0 to 12.0 cm. Giant EPs, although rare, are clinically significant because of their large size and frequent association with symptoms. A heightened suspicion of malignancy may lead to unnecessary diagnostic interventions and overtreatment. However, the overall prevalence of malignancy within EPs remains low, ranging from 1 to 3% (23). Most giant EPs are associated with postmenopausal tamoxifen therapy (24). In this study, all fifteen cases involved in postmenopausal women with no history of hormonal exposure, and histopathological examination confirmed benign pathology in every sample. Therefore, preoperative differentiation between benign and malignant lesions is crucial for guiding appropriate surgical management and avoiding overtreatment.

The development of EPs is attributed to a complex interplay of molecular mechanisms. These include local estrogen excess driven by aromatase overexpression, a disrupted estrogen–progestin balance, evasion of apoptosis, genetic alterations promoting clonal expansion, and inflammation-associated cellular signaling (25). Typically, giant EPs are predominantly associated with unopposed estrogenic exposure, notably among women undergoing tamoxifen treatment (24). Larger EPs have been shown to be risk factors for premalignant or malignant pathology (25). In this study, histopathological analysis of the resected specimens revealed benign findings in the majority of cases (11/15), whereas four cases were malignant (comprising two carcinomas, one carcinosarcoma, and one sarcoma). This proportion is notably higher than the overall prevalence of malignancy among EPs reported in the literature, which typically ranges from 1 to 3% (23). Therefore, given the elevated risk of malignancy observed in this study, giant EPs necessitate immediate diagnostic evaluation.

In the initial evaluation of PMB, TVS serves as the first-line noninvasive modality (26). Fleischer et al. screened 1,750 asymptomatic postmenopausal women and reported that an endometrial thickness ≤ 6 mm had a negative predictive value of 99.94% for excluding endometrial malignancy, reinforcing the utility of TVS in risk stratification (27). In contrast, blind endometrial biopsy or D&C suffers from low diagnostic accuracy and is not recommended. For definitive diagnosis and management, hysteroscopic polypectomy is the gold standard for identifying giant EPs, offering direct visualization and complete specimen retrieval for histopathological assessment.

Nevertheless, four patients with benign pathology were treated with TAH-BSO as their initial surgical intervention. Case 1 involved a patient with obesity, hypertension, and diabetes and featured the largest polyp (12.0 cm in diameter) (11). In case 2, TAH-BSO was performed because of preoperative suspicion of endometrial cancer; however, the lack of high-risk imaging features or histopathological findings justified such an aggressive surgical approach (12). In case 12, the lesion was misdiagnosed as a submucosal fibroid on TVS, leading to unnecessary hysterectomy (14). Similarly, in case 14, the patient was managed with TAH-BSO solely on the basis of imaging findings suggestive of an endometrial polyp, without prior hysteroscopic evaluation or endometrial biopsy (10). Collectively, these four cases illustrate that a definitive diagnosis was not established preoperatively, and all patients underwent radical surgery that could have been avoided with the use of minimally invasive diagnostic and therapeutic strategies. Therefore, the excision of EPs is indicated not only for symptom control and suspicion of malignancy, but also for assessing their intrinsic malignant potential. Given its high diagnostic yield, therapeutic efficacy, and minimal morbidity compared with hysterectomy, hysteroscopic polypectomy remains the cornerstone of surgical management for giant EPs.

## Strengths and limitations

This study presents one of the largest reported case series of giant EPs in postmenopausal women without hormonal exposure, comprising fifteen well-documented cases including one from our institution and fourteen identified through a systematic literature review. However, some limitations should be acknowledged. First, the sample size remains small because of the rarity of giant EPs in this population. Second, variability in the quality and completeness of reporting across published cases may introduce information bias. Despite these limitations, this series provides valuable insights into the clinical behavior and optimal management of giant EPs in postmenopausal women, supporting their generally benign nature while underscoring the need for complete excision and pathological evaluation.

## Conclusion

Giant EPs are rare but represent a notable cause of PMB. TVS is the first-line imaging modality for initial evaluation. Despite their size, these giant polyps are predominantly benign in the absence of hormonal exposure. Diagnostic hysteroscopy with complete polypectomy is the gold standard, enabling accurate histopathological assessment and avoiding unnecessary hysterectomy. In addition, hysterectomy in postmenopausal women with giant EPs should be reserved for cases with confirmed malignancy or other compelling clinical indications, rather than polyp size alone.

## Data Availability

The raw data supporting the conclusions of this article will be made available by the authors, without undue reservation.
